# Nano GO Consortium—A Team Science Approach to Assess Engineered Nanomaterials: Reliable Assays and Methods

**DOI:** 10.1289/ehp.1306866

**Published:** 2013-05-06

**Authors:** Thaddeus T. Schug, Srikanth S. Nadadur, Anne F. Johnson

**Affiliations:** 1 Cellular, Organs and Systems Pathobiology Branch, Division of Extramural Research and Training, National Institute of Environmental Health Sciences, National Institutes of Health, Department of Health and Human Services, Research Triangle Park, North Carolina, E-mail: nadadurs@niehs.nih.gov; 2 MDB Inc., Research Triangle Park, North Carolina

Two articles in this issue of *Environmental Health Perspectives*—Xia et al. (2013) and Bonner et al. (2013)—report results of a unique collaborative approach to environmental health research. The consortium behind these studies (the Nano GO Consortium), which is developing standardized methods for assessing the health and safety implications of engineered nanomaterials (ENMs), represents a new model of shared science that may offer lessons for other emerging and fast-evolving areas of research.

ENMs (man-made particles with any external dimension between 1 and 100 nm) have enabled considerable advances in electronics, drugs and medical devices, environmental remediation, and many other areas (Kessler 2011). They are fast becoming ubiquitous in products such as sunscreens, cosmetics, clothing, and building materials. Global demand for nanomaterials and nanoenabled devices is expected to approach $3.1 trillion by 2015 (Marquis et al. 2009).

As the production and use of ENMs grows, there are increased opportunities for ENMs to interact with biological systems. The physical, chemical, and biological characteristics of ENMs that make them useful for so many applications also make them potentially hazardous for living systems. The unique properties stemming from their small dimensions, such as high reactivity, large surface area, and the tunable nature of their optical, electrical, and magnetic properties, differentiate them from other materials in fundamental ways. The vast and expanding array of ENMs entering the environment could present health risks to researchers, workers, and consumers.

Although researchers have made progress in understanding biological responses to nanomaterials, the risks of exposure are not sufficiently understood to allow development of science-based risk assessment guidelines to support regulatory decision making (Maynard et al. 2006). There is a need to examine nanomaterial exposure, absorption, distribution, metabolism, and excretion and to relate ENM properties to biological responses at the cellular, tissue, and whole-organism level.

Determining the health effects of ENMs presents some unique challenges. The thousands of ENMs in use today are made from an enormous range of substances, vary considerably in size, and take a diversity of shapes, including spheres, cubes, cones, tubes, and other forms. They are also produced in different laboratories across the world using a variety of methods. In the scientific literature, findings on the properties and toxicity of these materials are mixed and often difficult to compare across studies. To improve the reliability and reproducibility of data in this area, there is a need for uniform research protocols and methods, handling guidelines, procurement systems, and models.

Because ENMs behave as particles, their size, shape, agglomeration state, surface chemistry, reactivity, and surface area, along with the number of particles, are all important parameters of ENM dose and are therefore factors in determining the ENM dose–response relationship. As a result, full and accurate characterization of the physical and chemical properties of ENMs is critical in assessing the biological effects of these materials. However, most of the published research does not provide detailed physical and chemical characterization of the nanomaterials or identify which characteristics are instrumental in their biological effects. New risk assessment models are needed that incorporate detailed characterization of ENMs with mechanistic and systems-level effects.

Although there is still much to learn about the toxicity of ENMs, we are fortunate to start with a clean slate: There are as yet no documented incidences of human disease due to ENM exposure (Xia et al. 2009). Because ENMs are manmade rather than natural substances, we have an opportunity to design, manufacture, and use these materials in ways that allow us to reap the maximum benefits—and minimal risk—to humans.

With $13 million from the American Recovery and Reinvestment Act (2009), the National Institute of Environmental Health Sciences (NIEHS) awarded 13 2-year grants to advance research on the health impacts of ENMs (NIEHS 2013). Ten grants were awarded through the National Institutes of Health (NIH) Grand Opportunities program and three were funded through the NIH Challenge Grants program. One goal of this investment was to develop reliable, reproducible methods to assess exposure and biological response to nanomaterials.

To advance their shared goal, the grantees were brought together to form the Engineered Nanomaterials Grand Opportunity (Nano GO) consortium. Within the framework of the consortium, grantees designed and conducted a series of “round-robin” experiments in which similar or identical methods were used to perform *in vitro* and *in vivo* tests on the toxicity of selected nanomaterials concurrently at 13 different laboratories. The consortium quickly became an active hub of communication. Four in-person meetings, combined with conference calls held at least bimonthly, allowed consortium members to work closely together to share experiences and results, identify problems, and refine methods and protocols as the tests proceeded. These experiments and their results are described by Xia et al. (2013) and Bonner et al. (2013).

The findings of the round-robin studies elucidate some important biological interactions of ENMs and can help inform the development of predictive models needed to establish regulatory guidelines for ENM production and use. Beyond the specific outcomes related to the toxicity of the tested nanomaterials, however, several broader lessons can be drawn from the structure of the Nano GO Consortium and the research process itself.

First, the process of working together to design and conduct concurrent round-robin experiments was crucial to the participants’ ability to develop reliable, reproducible research methods and protocols for studying the health effects of ENMs. Frequent communication among principal investigators and laboratory technicians allowed consortium members to continuously compare, troubleshoot, and refine protocols, ultimately significantly improving the harmonization of results. In addition, centralized characterization of the selected ENMs proved critical to researchers’ ability to standardize approaches and interpret results across laboratories. The NIEHS Centers for Nanotechnology Health Implications Research consortium, launched in 2011, will continue to build upon these protocols.

Second, the consortium structure motivated participants to communicate much more openly than is typical in most areas of research. In a discussion session held during the consortium’s final meeting, several participants noted that the shared goal of harmonization of methods and results across laboratories encouraged participants to fully engage with the planning process and to scrupulously follow the protocols to avoid being an outlier. In addition, repeating the same experiments in different laboratories proved scientifically valuable because researchers were able to have more confidence in the results.

Finally, several participants identified interdisciplinary collaboration as a key benefit of the consortium. Drawing upon participants’ diverse collective knowledge allowed the group to effectively tackle complex problems that would have been much more difficult for a single laboratory or researcher to resolve alone. In addition, some participants reflected that interdisciplinary exchanges helped them achieve unexpected advances that will be valuable in moving the field forward.

Conducting experiments in a round-robin format within a consortium structure is an unfamiliar approach for most researchers. Although some researchers acknowledged that working collaboratively with such a large and diverse group at times stretched the limits of their comfort zones, the consortium ultimately proved to be “greater than the sum of its parts,” resulting in reliable, standardized protocols that would have been difficult for researchers to achieve by working independently. Indeed, many participants reflected that participating in the consortium not only benefitted their shared goals but also enhanced their individual research efforts. The round-robin approach and the overall consortium structure may be valuable models for other emerging areas of science.
